# Vaccine Adjuvants Derived from Marine Organisms

**DOI:** 10.3390/biom9080340

**Published:** 2019-08-03

**Authors:** Nina Sanina

**Affiliations:** Department of Biochemistry, Microbiology and Biotechnology, School of Natural Sciences, Far Eastern, Federal University, Sukhanov Str., 8, Vladivostok 690091, Russia; sanina.nm@dvfu.ru; Tel.: +7-423-265-2429

**Keywords:** squalene, cucumariosides, chitosan, fucoidans, carrageenans, laminarin, alginate

## Abstract

Vaccine adjuvants help to enhance the immunogenicity of weak antigens. The adjuvant effect of certain substances was noted long ago (the 40s of the last century), and since then a large number of adjuvants belonging to different groups of chemicals have been studied. This review presents research data on the nonspecific action of substances originated from marine organisms, their derivatives and complexes, united by the name ‘adjuvants’. There are covered the mechanisms of their action, safety, as well as the practical use of adjuvants derived from marine hydrobionts in medical immunology and veterinary medicine to create modern vaccines that should be non-toxic and efficient. The present review is intended to briefly describe some important achievements in the use of marine resources to solve this important problem.

## 1. Introduction

The oil-in-water-based complete Freund’s adjuvant developed by Jules Freund and Katherine McDermott in 40s of last century is the first vaccine adjuvant. The basis of immune stimulation and provide immunologists with a way to stimulate the production of antibodies and cellular immune responses to weak antigens. This elaboration allowed to establish the basis of immune stimulation and provide immunologists with an instrument to stimulate the production of antibody and cellular immune responses to weak antigens. Today, adjuvants are of primary importance in vaccination strategies.

Throughout its history, mankind has fought with infectious diseases and made different scientific discoveries in the field of studying pathogens and the mechanisms of their effects on the host organism. These efforts resulted in the emergence of vaccinology based on the achievements of immunology, microbiology, biochemistry, and other sciences. Due to vaccination, most acute socially significant infectious diseases—such as smallpox, polio, tetanus, measles, diphtheria, and rabies—are practically eliminated or controlled [1]. That is perhaps one of the outstanding medical achievements [2]. Vaccines allow to save millions of people from illness, disability, and death each year. The World Health Organization (WHO) evaluates vaccination as one of the most cost-effective method of fighting for the preservation of human and animal health in many countries around the world [3].

Most vaccines that are successfully used today are based on inactivated (killed) or live attenuated (weakened) whole pathogens (viruses or bacteria). However, these empirically designed vaccines may exhibit undesirable side effects [4]. For example, the so-called killed vaccine may not be sufficiently inactivated, and weakened vaccines may reverse to a wild strain or cause a related disease in people with immunodeficiency or impaired health. When vaccines are inactivated, epitopes important for a protective response can be destroyed. Moreover, microorganisms may contain antigens that can cause such an undesirable response as the formation of blocking antibodies, which prevent the binding of functional, bactericidal antibodies [5]. Finally, traditional approaches to vaccination were unsuccessful against many important pathogens [6].

Developing safer vaccines is needed an approach based on current knowledge of the mechanisms involved in protective immune responses [7]. The most promising trend in rational design is the creation of subunit vaccines which, unlike traditional ones based on the whole pathogen, contain microbial antigen/s determining the development of a protective immune response in the macroorganism [8].

However, individual antigens usually induce a weak immune response, insufficient to protect the organism. To enhance immunogenicity, antigens must be presented to the immune system appropriately. Various adjuvants can be used for this purpose. Adjuvants (from the latin ‘*adjuvare*’, which means ‘to help’) are substances of different origin and composition that improves the effectiveness of vaccines through their effect on the innate immune system. In turn, these nonspecific responses influence the adaptive immune response, enhancing and modulating the specific immune response to vaccine antigen [9].

Oceans are the habitat of organisms characterized by great diversity. Marine hydrobionts are abundant in different biomedical active substances of different chemical structures [10]. Some of them possess adjuvant properties. The present review summarizes the most important achievements in the development of vaccine adjuvants based on substances isolated from marine organisms.

## 2. Adjuvant Families

### 2.1. Squalene

**Figure 1:**
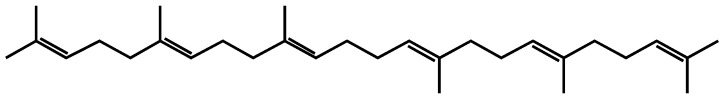
Structural formula of squalene.

Squalene (2,6,10,15,19,23-hexamethyltetracosa-2,6,10,14,18,22-hexaene, C_30_H_50_) is unsaturated triterpene, consisting of six isoprene groups (Figure 1) [11]. 

Initially, squalene has been widely used in cosmetics due to its emollient, moisturizing, and antioxidant effects on the skin. Then, squalene has also been used as an adjuvant in vaccines against some virus diseases. The Japanese scientist Mitsumaro Tsujimoto was the first to discover squalene which was isolated from the liver of the shark *Deania hystricosa* in 1906 [12]. The obtained substance was called "squalene" due to the name of a family of sharks Squalidae. In 1936, the study was continued by Nobel laureate Paul Karrer, who described in detail the molecular structure of squalene. The discovery of a naturally occurring substance—squalene was a new stage of creating nonspecific stimulators. Squalene used in vaccines is obtained from fish oil, and in particular shark liver oil following additional purification, due to the high level of squalene in sharks compared with other sources. The concentration of pure squalene in shark liver can reach about 80%. The main source of commercially obtained squalene is the most abundant species of shark known as the spiny dog fish *Squalus acanthias*. Alternative sources of squalene, including olives are known [13], while they are not established for commercial production. Marine yeast strains JCC207 and SD301 belonging to *Pseudozyma* species also produce large amount of squalene. Therefore, these novel strains are considered as the best candidate for the commercialization of microbial squalene [14,15].

Unlike pure squalene, which does not possess adjuvant properties, its emulsions with surfactants enhance the immune response. Biodegradable/biocompatible squalene is a component of the currently most-used adjuvant MF59^®^ which belongs to Novartis, as patented water-in-oil emulsion. The MF59^®^ also contains two stabilizing nonionic surfactants Tween 80 and Span 85, which are sourced from plants [16]. So, both detergents are also biodegradable substances. The emulsion causes an inflammatory reaction and mainly acts on macrophages and dendritic cells in the injection site. MF59^®^ increases the absorption of antigens by monocytes and promotes their migration to the lymph nodes. Compared to aluminum salts, MF59^®^ causes stronger immune response, stimulating both antibody production and T-cell immune response [17].

The adjuvant MF59^®^ was developed as a result of solving a dilemma on how to create adjuvant which is safe similar to aluminum salts, but possesses a strong immunostimulating activity similar to Freund's complete adjuvant based on a water-in-mineral oil emulsion [11]. MF59^®^ is included in the Fluad^®^ influenza vaccine, originally licensed by Chiron Corporation (1997) to be used in the elderly, and recently in infants and young children also. Fluad^®^ is now licensed in 30 countries worldwide [11,18]. Another licensed influenza vaccine, Pandemrix™, is adjuvanted with a system AS03 composed of the same quantity of squalene (∼2% (*v/v*)) as well as the immune potentiator DL-α-tocopherol (vitamin E) and a surfactant polysorbate 80 (polyoxyethylene sorbitan-20 monooleate) in an oil-in-water emulsion [19]. After the 2009–2010 H1N1 pandemic vaccination, Pandemrix™ began to be associated with narcolepsy. It turned out that the adjuvant AS03 is not the cause of this rare disease, while the etiology of narcolepsy is still unclear [20,21].

In spite of the safety and high effectivity of MF59^®^, unfounded claims were made against this adjuvant in the 90s. The so-called Gulf War syndrome was connected with antibodies to squalene which were found in the blood of veterans after administration of the anthrax vaccine. It has been proven that squalene was not contained in these vaccines. It has also been found that antibodies to squalene are found in the majority of the adult population, regardless of whether they received a vaccine containing squalene. Antibody levels against squalene increase with age [11,22].

Squalene-based adjuvant GLA-SE is a stable oil-in-water emulsion (SE) combined with glucopyranosyl lipid adjuvant (GLA) which is a synthetic agonist of toll-like receptor (TLR) 4. This adjuvant in combination with a clinical candidate antigen ID93, which is a fusion of four *Mycobacterium tuberculosis* proteins, induced multifunctional CD4+ Th1 cell responses and significant protection against tuberculosis in laboratory animals [23,24]. The recombinant malaria antigen PfCelTOS combined with GLA-SE also stimulates strong Th1-type immune responses in mice [25].

Squalene is also included in the content of the second generation adjuvants, CAF19 and CAF24, which belong to so-called cationic adjuvant formulation (CAF) family adjuvants initially created due to a need for new safe adjuvants that could induce a strong Th1 response [26].

### 2.2. Saponins

Saponins are naturally occurring glycosides containing an aglycone unit linked to one or more carbohydrate chains. The aglycone moiety consists of a sterol or the more common triterpene part. This chemically heterogeneous group of glycosides possesses diverse biological activity including immunostimulating properties [27,28] that exert a wide range of health benefits and provokes considerable interest in these substances. Saponins were first discovered in higher plants where they are widely spread [29]. The QS-21 saponin which is a mixture of two regioisomers of soluble triterpene glycosides isolated from the South American tree *Quillaja saponaria* Molina has been shown potent adjuvant activity. However, QS21 is not used for human vaccines mainly due to its toxicity [30]. As a result of the search for new pharmacologically active substances, saponins were also detected and isolated from marine organisms such as sea cucumbers [31], starfish [32], sponge [33], alga [34,35], and coral [36].

Saponins are the most important and abundant secondary metabolites of holothurians, commonly known as sea cucumbers (the class Holothuroidea of the Echinodermata phylum). The immunostimulating activity of saponins from holothurians in relation to mammals was first demonstrated on the example of crude holothurin, which was a mixture of saponins isolated from cuvierian glands of the sea cucumber *Actinopyga agassizi*. These compounds at concentrations of 4–100 µg per mL leucocyte suspension stimulated in vitro the phagocytosis of *Staphylococcus aureus* by polymorphonuclear leucocytes of human blood [37].

Holothurins usually belong to triterpene glycosides which are abundantly present in sea cucumbers [38]. The majority of triterpene glycosides or triterpene saponins from sea cucumbers belong to the holostane series having 3β-hydroxy-5α-lanostano-γ(18, 20)-lactones in aglycone. Their carbohydrate part is composed of two to six monosaccharide residues and may have from one to three sulfate groups [31]. Triterpene glycosides from holothurians have a wide range of biological activities including immunomodulating, immunoadjuvant, and anti-inflammatory activities. The results of numerous studies show the ability of triterpene glycosides of holothurians to strengthen non-specific immunity by enhancing the processes of phagocytosis [39].

**Figure 2:**
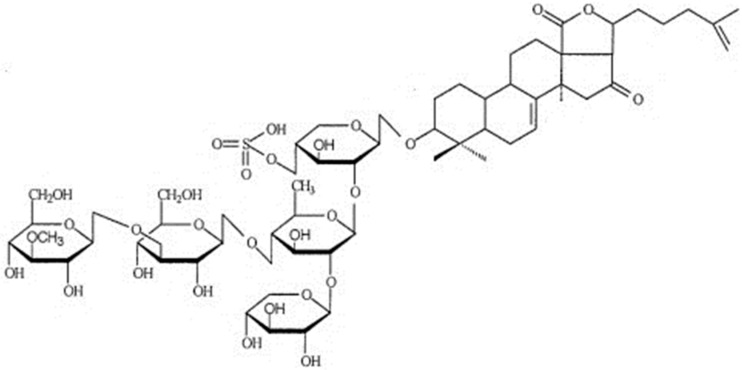
Structural formula of triterpene glycoside cucumarioside A_2_-2.

Cucumariosides from the far-eastern edible sea cucumber (holothurian) *Cucumaria japonica* have a pronounced adjuvant effect increasing the level of antibodies under the action of the particulate pertussis vaccine, as well as enhancing the protective effect of the vaccine [40]. Triterpene glycoside cucumariside A_2_-2 (Figure 2), isolated from *C. japonica*, at nanomolar concentrations, has immunostimulating activity, which is expressed primarily in the activation of cellular immunity. This effect was appeared to relate with purine receptors of the P2X family (P2X1 and P2X4 types), which provide ATP-dependent Ca^2+^ transport through the membranes of macrophages. Cucumarioside A_2_-2 as an allosteric modulator of purine receptors, binding to them, can activate the abrupt and reversible entry of calcium ions into cells from the extracellular space that results in the enhancement of cellular immunity [41].

Triterpene glycosides of holothurians are typical membranotropic substances [42] due to the high affinity for cholesterol—i.e., the ability to form irreversible glycoside-sterol complexes of a different morphology [43]. In combination with cholesterol, the triterpene glycosides of holothurians are non-toxic and can act as carriers of various hydrophobic or amphipathic antigens. In this regard, triterpene glycosides from commercial sea cucumber *C. japonica* are of particular importance.

Cucumarioside A_2_-2 revealed the best capacity to form tubular supramolecular complexes with cholesterol in comparison with other related triterpene glycosides of holothurians [44]. Therefore, this saponin was applied to construct nanoparticulate antigen delivery systems with adjuvant activity, which was named tubular immunostimulating complexes (TI-complexes) [45,46]. Beyond cholesterol and cucumarioside A_2_-2, TI-complexes comprise monogalactosyldiacylglycerol (MGDG) from marine algae or seagrass, which form lipid matrix for incorporation of protein antigen [47]. TI-complexes are able to stimulate immune response both to the membrane [48] and to water-soluble proteins [49]. Their adjuvant effect to antigens depends on the physicochemical properties of MGDG [50] and chemical structure of saponin [49].

A structural diversity of triterpene glycosides from sea cucumbers is determined by different features, in particular, by the occurrence of acetoxy groups in the aglycone and number and position of sulfate groups in the sugar moieties. Acetylated saponins are mainly found in the family Cucumariidae, whereas they are very rare in the genus Holothuria [51]. The first acyl (acetoxy) group of acetylated saponins usually is located at C-16 of aglycones. Sulfated triterpene glycosides contain a sulfate group at the C-4 of the first xylose. Some acetylated compounds also contain a sulfate group bonded to their sugar residues.

Acetylated saponins isolated from sea cucumbers show strong immunomodulatory activity. For instance, frondoside A, which is the major triterpene glycoside of the sea cucumber *C. frondosa*, stimulated cell-based immunity and very weakly influenced the humoral immune response [52] in contrast to Cumaside (a non-toxic complex of monosaulfated glycosides mainly cucumarioside A_2_-2 from *C. japonica*) which has a remarkable stimulatory effect on the humoral immune system [53]. Monosulfated triterpene glycosides isolated from *C. japonica* were described as the most effective immunostimulants in contrast to di- and trisulfated saponins which were reported as immunosuppressors [54]. Monosulfated triterpene glycosides okhotosides B_1_, A_1_-1, A_2_-1, and especially frondosides A, A_1_, and cucumarioside A_2_-5 isolated from *C. okhotensis* [53] as well as cucumariosides I2, A5, and B2 isolated from *Eupentacta fraudatrix* also increase lysosomal activity of mouse macrophages [55].

### 2.3. Chitosan

**Figure 3:**
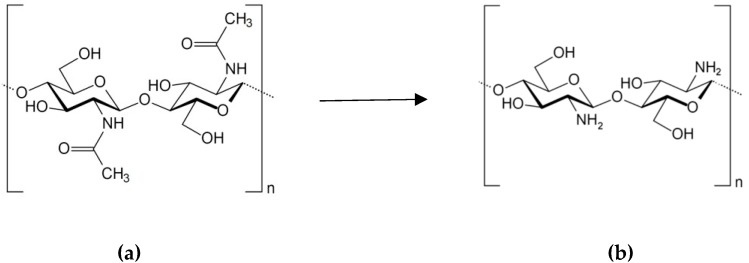
Transformation of chitin (**a**) to chitosan (**b**) by deacetylation.

A linear polysaccharide chitosan is a random copolymer of d-glucosamine and *N*-acetyl-d-glucosamine linked by β-(1→4) bonds. While the major monomer unit of chitosan is d-glucosamine. Chitosan is a product of partial deacetylation of biopolymer chitin (Figure 3), which is the second most common polysaccharide in nature, after cellulose. Chitin is found mainly in the exoskeleton of oceanic crustaceans (crabs, shrimps, lobsters, and krill), as well as in the cuticle of insects, the cell wall of fungi and bacteria. However, the main types of raw materials for commercial production is chitin from crustacean shell waste. Thus, the production of chitin and chitosan is an example of rational use of natural resources and solving environmental problems by expanding the use of biodegradable biopolymers.

Chitin is a long-chain polymer of *N*-acetyl-d-glucosamine linked by β-(1→4) bonds. During deacetylation of chitin, acetyl groups are partially removed. The incomplete conducting of the chitin deacetylation reaction causes the structural heterogeneity inherent in chitosan where degree of deacylation reaches of 65–70% and above. The distribution of the residual N-acetylated units along the polymer chain can significantly affect some physicochemical properties and biological activity of chitosan. Deacetylation of chitin also results in depolymerization reaction, revealed by changes in molecular weight (MW) of chitosan [56]. Chitosan is poorly soluble in water, but, unlike chitin, it dissolves in dilute organic and mineral acids. In acid medium, the large amount of free amino groups appears in chitosan, which behaves like a cationic polyelectrolyte and therefore is of great commercial interest. It is the only natural polysaccharide exhibiting a cationic character responsible for some unique properties [57].

Chitosan is non-toxic, biocompatible, biodegradable biomaterial, possessing antitumor, antioxidant, antimicrobial, and immunomodulating properties that makes it very attractive for a variety of biomedical applications [58]. Chitosan has potential vaccine adjuvant properties that were first reported by Nishimura K. and coauthors in 1984–1985 [59]. Later, different forms of chitosan (solutions, gels, powders, micro- and nanoparticles, multicomponent formulations for parenteral, and mucosal administrations) were used successfully as vaccine adjuvants [60].

The adjuvant properties of chitosan in the content of mucosal vaccines are based on mucoadhesive properties of this cationic polyelectrolyte. Due to positive charge, chitosan is able to interact with negatively charged mucosal surface that offers various advantages compared with conventional parenteral administration of vaccines such as non-invasive, needle-free injection, induction of both systemic and mucosal immune responses. Free amino groups also are responsible for controlled antigen release, transfection, enhancement of permeation, and efflux pump inhibitory properties. Chitosan is an effective transmucosal absorption promoter that is especially important for vaccine delivery [61,62].

**Figure 4:**
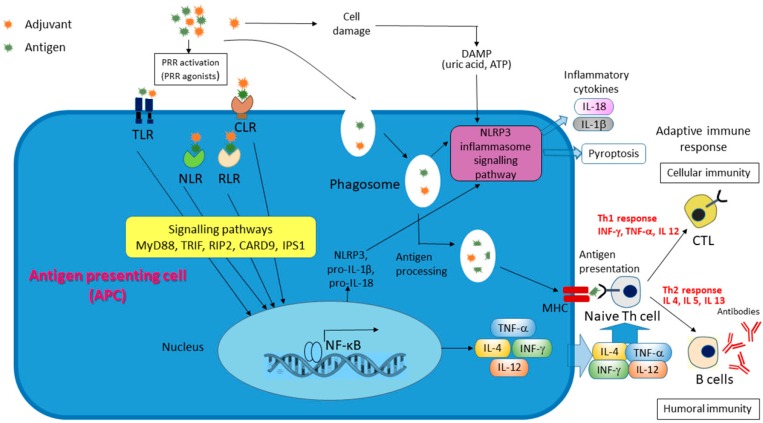
Putative mechanisms of action of the proposed adjuvants. Pattern recognition receptors (PRRs) allow innate immunity cells to recognize pathogen-associated molecular patterns (PAMPs) of microbes or damage-associated molecular patterns (DAMPs), which are released from injured or dying cells. The PRRs includes Toll-like receptors (TLRs), Nod-like receptors (NLRs), and и C-type lectin receptors (CLRs), which are considered receptors of adjuvants. NLRP3—NLR pyrin domain-containing protein 3.

Mucosal vaccines adjuvanted with chitosan have induced strong antibody and T-cell responses [63,64]. As shown, chitosan is phagocytosed by macrophages that potently activates the Nod-like receptor (NLR) pyrin domain-containing protein 3 (NLRP3) inflammasomes and may causes adjuvant activity of chitosan [65] (Figure 4). This effect of chitosan is size-dependent: small particles possessed the greatest activity. Chitin revealed only a very weak stimulation, and soluble chitosan did not activate inflammasomes. TLR4, CD14, and the mannose receptor are involved in the recognition of chitosan by innate immune cells [66], whereas chitin is sensing by TLR2, Dectin-1, and the mannose receptor [67]. Recently, it was shown that chitosan activates dendritic cells and promotes Th1 responses by involving the DNA sensor cGAS-STING pathway [68,69].

To improve solubility and widen medical applications, chitosan is modified by acylation, alkylation, sulfation, hydroxylation, etc. [58,70]. Micro- and nanoparticles of chitosan and its derivatives are the most preferable form of vaccine adjuvants and delivery vehicles [71,72,73]. Microspheres obtained from chitosan are more effective adjuvants for different antigens compared with commonly used microspheres from polylactic acid (PLA) and poly(lactic-co-glycolic) acid (PLGA), since chitosan microspheres provide a controlled release rate of captured antigens [71]. As a result, the need for repeated administrations, which is the main drawback of some currently available vaccines, is eliminated.

### 2.4. Seaweed Polysaccharides 

Seaweed polysaccharides are known as the immune regulators which could activate the immune cells and promote homeostasis in organism. Polysaccharides, which function as adjuvants, seem to have promising effects for the targeted immunity stimulation. Among them, the sulfated polysaccharide has attracted much attention [74].

Fucoidan, or fucoidans, is a group of various fucose-rich sulfated polysaccharides, whose chemical structure depends on the source species, environment of the source seaweeds, extraction parameters, etc. [75]. Fucoidans are mainly extracted from the brown algae of the orders Laminarales and Fucales. The polysaccharides isolated from Fucales are characterized by alternating α-(1 → 3)- and α-(1 → 4)-glycosidic bonds between fucose residues in the main chains, whereas these residues are linked by α-(1 → 3)-glycosidic bonds only in fucoidans from Laminarales [76]. Fucoidan was shown to have stimulatory effects on macrophages, dendritic cells and other types of immune cells. The recent studies described the application of fucoidan from *Fucus vesiculosus* and *F. evanescens* as a vaccine adjuvant. Killed *Bordetella bronchiseptica* and *Mycoplasma hyopneumoniae*, which are causative pathogens of swine respiratory diseases and swine enzootic pneumonia, respectively, are commonly administered as vaccine antigens in veterinary clinics. Co-administration of *B. bronchiseptica* antigen and fucoidan from *F. vesiculosus* increased production of pro-inflammatory cytokine, tumor necrosis factor-α (TNF-α) by spleen cells. Also, fucoidan stimulated the production of antigen-specific antibodies in mice immunized with *M. hyopneumoniae* antigen. In general, the results of this study allowed to get useful information on the adjuvant properties of fucoidan [77]. Fucoidan from *F. evanescens* stimulated the formation of specific antibodies to the surface antigen of the hepatitis B virus (HBsAg) and increased the level of the pro-inflammatory cytokines (TNF-α, interferon γ (IFN-γ), interleukin 2 (IL-2)). The adjuvant effect of fucoidan and its structural modifications was comparable to that of the traditional licensed adjuvant aluminum hydroxide. The obtained results indicate a promising use of sulfated polysaccharides from *F. evanescens* as vaccine adjuvants [78]. However, the human body is difficult to absorb focoidans due to the high MW of polysaccharides. Therefore, immunostimulating activity of the low-molecular-weight (LMW) fucoidans was studied in some recent works. Orally administrated the LMW fucoidan (MW lower than 3 KDa) from brown seaweed *Laminaria japonica,* which was rich in fucose and sulfate stimulated splenocyte proliferation, NK cell activity, and phagocytic activity, as well as IL-2, IL-4, and IFN-γ secretion. In addition, the LMW fucoidan enhanced antigen-specific antibody production in ovalbumin- and *Mycoplasma pneumoniae* antigen-stimulated mice. Therefore, the immunostimulating ability of LMW fucoidan, a natural dietary supplement, could contribute to reducing the *M. pneumoniae* infectious disease [79]. Similar results were received with the LMW (MW < 10 kDa) fraction of fucoidan from the brown alga *Undaria pinnatifida* which possessed the ability to promote synthesis of nitric oxide (NO), TNF-α, and IL-6 by activating the nuclear factor-kappa B (NF-κB) and a mitogen-activated protein kinase (MAPK) signaling pathways in RAW264.7 macrophages [80]. This was in agreement with previous studies [81,82]. However, attempts to establish a reliable correlation between the structure and biological activity of fucoidan fails, because their structural diversity is not yet fully investigated [76]. Other recent achievements in studies of the fucoidan effects on the immune system are summarized in [83].

The sulfated galactan from green algae *Codium fragile* showed stimulatory effect on NO production in murine macrophages and stimulated the induction of mRNAs of pro-inflammatory and anti-inflammatory cytokines. Therefore, authors suggested this polysaccharide may exhibit immunostimulating properties due to activation of macrophages [84]. Sulfated polysaccharides of other green algae *Caulerpa cupressoides* var. *flabellate* also demonstrated strong immunostimulatory activity. These polysaccharides were capable of increasing the production of inflammatory mediators by macrophages, which possess a significant role in enhancing both adaptive and innate immunity. The increased inducible nitric oxide synthase (iNOS) and cyclooxygenase-2 (COX-2) gene expression was also observed [74].

Carrageenans, high-molecular-weight sulfated and nonsulfated galactanes extracted from red seaweeds, consist of repeating galactose units and 3,6 anhydrogalactose joined by alternating α-1,3 and β-1,4 glycosidic bonds. Three widely used forms of carrageenans, κ-, ί-, and λ- differ by the number and position of the ester sulfate groups on the repeating galactose units [85]. In spite of a wide application of carrageenan in the food, cosmetics, and pharmaceutical industries, the data on immune properties of these polysaccharides are limited and controversial. However, recent data demonstrate their immunostimulating activity rather than immunosuppression effects [86]. It is known that carrageenans are able to activate macrophages, and induce the generation of pro-inflammatory cytokines and active forms of oxygen by cells of the immune system [87,88,89]. Сarrageenan (type unidentified) showed significant ability to enhance antigen specific immune responses as well as antitumor effects in mice immunized with human papillomavirus type 16 (HPV-16) E7 peptide vaccine. It was also demonstrated that the enhancement of the anti-E7 immune responses was generated by TLR4-mediated signaling pathway [90].

Non-gelling λ-carrageenan, which has three ester sulfate groups *per* every disaccharide unit, is used to create an inflammation model (rat paw edema) to study anti-inflammatory effects. Luo et al. reported about adjuvant activity of λ-carrageenan which significantly increased anti-cancer effects of vaccines [91]. Less sulfated κ-carrageenan is much less potent to induce rat paw edema than λ-carrageenan [92]. In turn, κ/β-carrageenan unlike other carrageenan forms is characterized by reduced content of sulfate groups and the highest capacity to evoke the synthesis of the anti-inflammatory cytokine IL-10 which inhibits the production of pro-inflammatory TNF-α [93].

The immunostimulating effect of carrageenan is also dependent on its MW. The LMW fractions (MW < 20 kDa) from the dominant ι-carrageenan extracted from red alga *Solieria chordalis* revealed potent ability to stimulate phagocytosis, proliferation of lymphocytes, cytotoxicity of natural killer cells, and antibody-dependent cell cytotoxicity. This was in agreement with the influence of the LMW fractions from carrageenans of κ- and λ-types [94].

Sulfated polysaccharide ulvan, found in *Ulva* spp. and mainly composed of sulfated rhamnose, uronic acids (glucuronic and iduronic acids) and xylose, also possesses immunostimulating activity [95].

Some red macroalgae synthesize other types of sulfated polysaccharides. Two xylomannan fractions of sulfated polysaccharides from the red seaweed *Nemalion helminthoides* induced in vitro proliferation of macrophages and significantly stimulated the production of inflammatory cytokines both in RAW 264.7 cells and in mice, whereas this response was not observed with the mannan fractions. Both xylomannan fractions also stimulated production of cytokines in vivo. These results demonstrated strong immunomodulatory properties of sulfated xylomannans [96]. Sulfated polysaccharide isolated from red algae *Porphyra haitanensis* showed similar immunostimulating effects [97].

Immunostimulating activity has been found for non-sulfated polysaccharides from seaweeds. laminarin, also called laminaran, is a LMW polysaccharide (MW < 10 kDa) found in large quantities in brown algae made up of β(1→3)-glucan with β(1→6)-branches [98]. It has been shown that this most studied glucan stimulates the activity of macrophages which strengthen the release of various inflammatory mediators. Immunostimulating activity of laminarin is carried out via the transcription factor pathway [99]. However, the strong stimulating effect of β-glucans on macrophages and neutrophils was first demonstrated on the example of β-glucan from yeast *Saccharomyces cerevisiae* many years ago. Microcapsules made of non-toxic β-glucan from *S. cerevisiae* (Adjuvax) as delivery system for antigens promoted over a 1000-fold increase of antibody level that was equivalent to the effect of complete Freund's adjuvant [100]. Now, β-glucans are extensively used to develop vaccine adjuvants [101]. Since the structures of β-glucans from fungi and algae are highly conservative [100], these polysaccharides found in seaweeds can also be considered as promising vaccine adjuvants.

**Figure 5:**
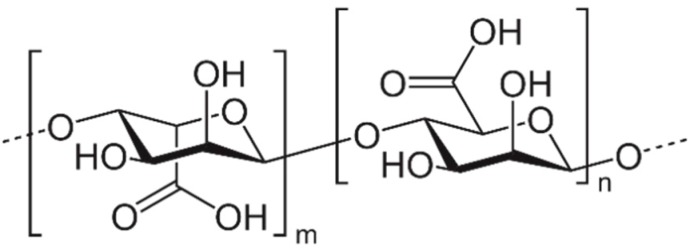
Chemical structure of alginate.

Other non-sulfated polysaccharide, alginate found in brown algae is a linear copolymer consisting mainly of repeated residues of β-d-mannuroniс acid (M) and α-l-galuronic acid (G) linked by α-(1→4)-glycosidic bonds (Figure 5). The monomers are linked together in different blocks: G-blocks, M-blocks, MG-blocks, or randomly organized blocks.

Soluble sodium alginate is widely used because of its biodegradability, biocompatibility, low toxicity, mucoadhesive nature, and a relatively low cost. The adjuvant activity of sodium alginate was demonstrated by the example of the Bacillus Calmette–Guérin (BCG) vaccine. The main disadvantage of this vaccine is the inability to stimulate an adequate T-cell response. Sodium alginate co-administered with the *Mycobacterium bovis* BCG vaccine, induced the two-fold enhancement of the protection level of mice against *M. bovis* due to significant stimulation of the specific anti-mycobacterial IgG production and the effective induction of the Th1 immune response judging by the increase in the IFN-γ production and the IgG2a/IgG1 ratio, associated with the Th1 and Th2 response, respectively [102]. Alginates are extensively applied to form hydrogel microspheres and nanospheres which are easily formed by ionic gelation technique that lead to the formation of calcium alginate complexes [103]. Soluble sodium alginates have shown less effect on immune response than the particulate form of alginate, promoting inflammatory cytokine production and activating dendritic cells [104]. Alginate nanoparticles loaded with the diphtheria toxoid showed about six times higher humoral immune response in immunized guinea pigs than conventional alum-based anti-diphtheria vaccine [105]. Alginate also is able to enhance adjuvant activity of micro/nanoparticles based on other polymers. The addition of small amount of alginate into PLGA matrix enhanced humoral and cellular immune responses against two malaria synthetic peptides, SPf66 and S3, encapsulated into PLGA–alginate microparticles. Alginate acted as a Th1 immunomodulator promoting a more balanced Th1/Th2 response [106]. Oral vaccine with plasmid-cured *Salmonella enterica* serovar Gallinarum, included in alginate-coated chitosan microparticles, induced higher expression of INF-γ and the same protection of chickens compared with the commercial fowl typhoid vaccine introduced parenterally [107]. Alginate-coated trimethylchitosan nanoparticles loaded with inactivated PR8 influenza virus generated a much higher IgG2a/IgG1 ratio than the non-coated ones and PR8 virus alone after intranasal administration [108].

## 3. Conclusions

The data summarized in the present review demonstrate both the successes already achieved with the use of some substances from marine hydrobionts as vaccine adjuvants, and the emerging prospects for using a large number of other components of marine organisms in this capacity (Table 1). 

Despite the promise of the described adjuvants, their low toxicity, high immunostimulating activity, and biological compatibility, only two of them are currently licensed for use in commercial vaccines. Moreover, the application of these adjuvants (MF59^®^ and AS03) is limited to their use in the content of anti-flu vaccines. The reason of this limitation is probably due to the fact that each adjuvant is intended for one or more antigens contained in vaccine. In the first case, the result obtained by using an adjuvant with one antigen cannot be extended to another antigen. Therefore, future research will be aimed not only at finding and licensing new immunostimulating substances, but also at expanding the spectrum of their use. In turn, it is necessary to know the mechanism of action of the proposed adjuvants, which is currently a weak point in spite of the progress achieved in the field of adjuvant development. Future adjuvants should comprise ligands for pattern recognition receptors (PRRs), in particular for Toll-like receptors (TLRs) and NOD-like receptors (NLRs) of innate immunity cells that can allow to regulate the Th1/Th2 polarization of the adaptive immune response, as well as accelerate and strengthen it (Figure 4). This is especially important if the recombinant protein antigen lacks a ligand for PRRs. Conventional needle injections (intramuscular, subcutaneous, and intracutaneous routes) that are associated with various adverse effects of adjuvants will be gradually replaced by needle free injections of the vaccines. This will increase the effectiveness of vaccination by delivering antigens to areas where there are sensory cells of innate immunity (mucous membranes, skin). This method is very promising for vaccination of children, the elderly, cancer patients, and persons with secondary immunodeficiencies. In this connection, more and more attention will be attracted to substances with mucoadhesive properties and/or capable of forming hydrogel microspheres and nanospheres, and nano-emulsions for mucosal and transdermal immunization, respectively. The growing attention of researchers to the problem of finding effective and safe adjuvants among substances of marine origin—which are distinguished by a large variety of chemical structures and biological activity—allows us to hope that new breakthrough results will appear that are necessary to solve an important global problem not only in medicine and health, but also in economics.

## Figures and Tables

**Table 1:** Advantages and disadvantages of adjuvants derived from marine organisms and licensed vaccines based on them.

Adjuvant Name	Advantages	Disadvantages	Licensed Vaccines
MF59^®^	Compared to aluminum salts, MF59^®^ causes stronger immune response, stimulating both antibody production and T-cell immune response.	Reactogenicity. Pain at injection site. Induces inflammatory arthritis.	MF59^®^ is included in the Fluad^®^ influenza vaccine which is now licensed in 30 countries worldwide.
AS03	The strong stimulation of both antibody production and Th1 and Th2 immune responses.	An association between the AS03-adjuvanted Pandemrix vaccine and narcolepsy cannot yet be excluded.	It is approved in the United States and Europe and used in various vaccine products. For example, in H1N1 pandemic flu vaccine Pandemrix and Arepanrix.
GLA-SE	Strong Th1-type immune responses. The enhancement of the magnitude and polyfunctional cytokine profile of CD4+ T cells.	Mild or moderate adverse events (mainly pain at injection site).	-
CAF19, CAF24	Promising adjuvant for induction of cytotoxic T lymphocytes (CTLs) responses upon intracutaneous and intramuscular immunization.	Specific lysis of antigen-pulsed splenocytes.	-
Cucumarioside A2-2	Immunostimulating activity expressed primarily in the activation of cellular immunity.	Individual form possesses membranotropic properties which is absent in complex with cholesterol.	-
Chitosan	Non-toxic, biocompatible, biodegradable, non-allergenic. Parenteral and mucosal administrations. Controlled antigen release. Mucosal administration elicits robust antibody and T-cell responses.	Poor reproducibility of the results due to the variability of the chemical structure. Poor solubility above pH 6.	-
Fucoidans	Almost complete absence of toxicity, safety, and excellent biocompatibility. Regulation of cellular and humoral immunity as well as hematopoietic mobilization. Potentiation of the function of immune cells. Anti-cancer effect.	Difficulties with obtaining structurally characterized and homogeneous samples or oligomeric fractions.	-
Carrageenans	No adverse side effects at intranasal use. An activation of macrophages. Induction of the generation of pro-inflammatory cytokines. Significant ability to enhance antigen specific immune responses as well as antitumor effects.	Limited solubility. Anticoagulant properties. Prolonged oral administration can lead to the development of inflammation of the gastrointestinal tract.	-
Alginate	Non-toxic, biocompatible, biodegradable. Mucoadhesive nature and a relatively low cost. Stimulation of Th1 response and production of specific antibodies. Anti-cancer and anti-allergic properties. An ability to form hydrogel microspheres and nanospheres which possess higher immunostimulating effect.	Variable chemical structures.	-
